# DNA damage response(DDR): a link between cellular senescence and human cytomegalovirus

**DOI:** 10.1186/s12985-023-02203-y

**Published:** 2023-11-01

**Authors:** Xinna Wu, Xuqiang Zhou, Sanying Wang, Genxiang Mao

**Affiliations:** 1 Affiliated Zhejiang Hospital, Zhejiang University School of Medicine, Hangzhou, 310030 China; 2 Zhejiang Provincial Key Lab of Geriatrics & Geriatrics Institute of Zhejiang Province, Department of Geriatrics, Zhejiang Hospital, Hangzhou, 310030 China; 3 College of Life Science, Zhejiang Chinese Medical University, Hangzhou, 310053 China

**Keywords:** Human cytomegalovirus, DNA damage, Cellular senescence, Senescence-associated secretory phenotype, Cell cycle

## Abstract

The DNA damage response (DDR) is a signaling cascade that is triggered by DNA damage, involving the halting of cell cycle progression and repair. It is a key event leading to senescence, which is characterized by irreversible cell cycle arrest and the senescence-associated secretory phenotype (SASP) that includes the expression of inflammatory cytokines. Human cytomegalovirus (HCMV) is a ubiquitous pathogen that plays an important role in the senescence process. It has been established that DDR is necessary for HCMV to replicate effectively. This paper reviews the relationship between DDR, cellular senescence, and HCMV, providing new sights for virus-induced senescence (VIS).

## Background

Cellular senescence was first formally described by Hayflick et al. over 50 years ago [1, 2]. Since then, it has been understood that cellular senescence is a stress-induced transformation in cellular states, including terminal cell cycle arrest and the development of senescence-associated secretory phenotypes (SASP) [3]. Senescence can be triggered by various types of cellular and environmental stresses such as telomere shortening, oncogene activation, oxidative stress, and DNA damage [4, 5]. Although many different factors lead to senescence, the DNA damage response (DDR) is a common factor in all of these mechanisms. Studies have demonstrated that senescence can be caused by persistent DDR [6, 7], a signaling cascade activated by DNA damage [5], in which cells respond to DNA damage by pausing cell cycle progression and trying to repair [7, 203].

Human cytomegalovirus (HCMV) is a β-herpesvirus that infects a variety of cell types, including fibroblasts, epithelial cells, macrophages, endothelial cells, dendritic cells, and smooth muscle cells [8]. As an enveloped, double-stranded DNA (dsDNA) virus, it has the largest genome of human viruses [9]. Herpesvirus genes are expressed in a “temporal cascade,” whereby the first set of viral genes, the immediate-early (IE) genes, drive the subsequent expression of delayed-early (DE) and late (L) genes [10–13]. During HCMV infection, the 72-kDa and 86-kDa IE1 and IE2 proteins are among the first and most widely expressed proteins. It is assumed that these proteins operate as transcriptional regulators by interacting with numerous cellular proteins that communicate with one another [14, 15].

A growing number of studies have shown that many viral infections, including HCMV [16–19], can also activate cellular senescence responses and that virus-induced senescence (VIS) has much in common with other forms of cellular senescence [20]. However, the precise regulatory mechanisms directly linking HCMV to cellular senescence remain unknown. As DDR signaling pathways are critical for the replication of HCMV [21–23], it would be interesting to investigate if HCMV can cause or worsen cellular senescence through DDR. In this review, we first provide a detailed explanation of how DNA damage response (DDR) begins and develops as well as how DDR contributes to the establishment of cellular senescence. We then concentrate on how HCMV influences DDR and ultimately causes cellular senescence which is characterized by the senescence-associated secretory phenotype (SASP).

## DNA damage response(DDR)

**Fig. 1:**
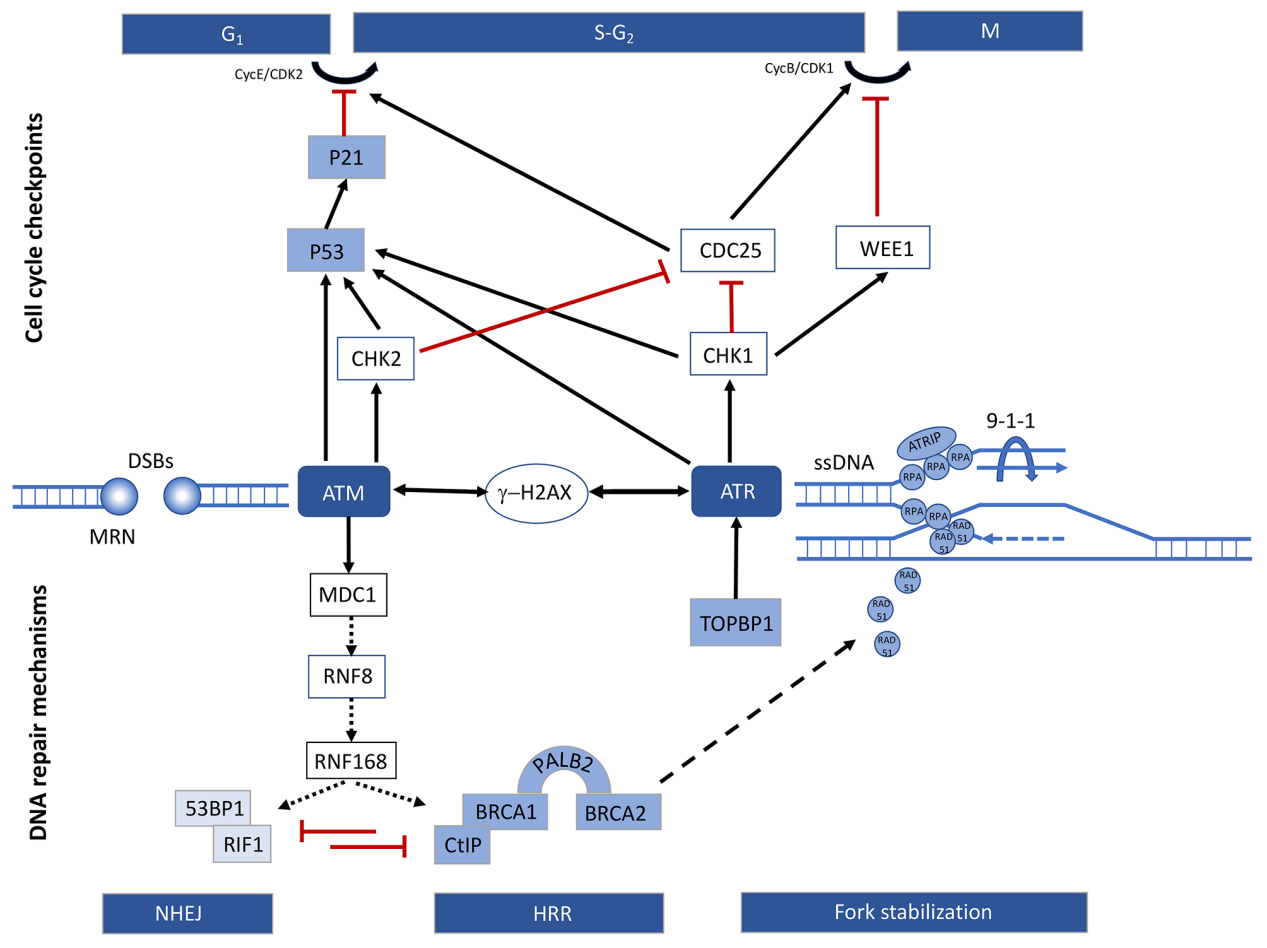
The DNA damage response. Responses to DNA damage caused by double-strand breaks (DSBs) and single-stranded DNA (ssDNA). The MRN complex detects DNA DSBs and signals them by activating ATM. The accumulation of ssDNA at stalled or stressed replication forks activates ATR. Following the activation of transducer kinases, DNA damage signaling is initiated, which includes DNA repair processes (lower panel) and cell cycle checkpoints (upper panel). Direct and indirect interactions are indicated by solid and dashed arrows, respectively. This figure was modified according to the published Fig. 1 in reference [203].

DNA damage activates a signaling cascade named DNA damage response (DDR) [5], in which cells respond to DNA damage by pausing cell cycle progression and trying to repair [7, 203] (Fig. 1). This complicated network of signaling channels made up of sensors, transducers, and effectors. The sensor delivers a signal to the transducer when it locates damaged DNA, such as DNA double-strand breaks (DSBs) or single-stranded DNA (ssDNA). The transducer amplifies the signal and transmits it to the effector. The effector executes a series of cellular responses, including initiating activation of cell cycle checkpoints and mobilizing the corresponding damage repair pathways [22]. If DNA damage is repaired in time, the cell will quickly return to normal; however, if the DNA damage is particularly severe and cannot be repaired, the cell may undergo apoptosis or cellular senescence. The former is programmed cell death, a form of cellular suicide that removes damaged cells from the cell population [24]; the latter is a natural irreversible cell cycle arrest, induced by DDR. It remains unclear what determines the choice between apoptosis and senescence, but determinants may include cell type and the intensity, duration, and nature of the damage [7].

The MRE11-RAD50-NBS1 (MRN) complex and the single-stranded DNA-binding protein replication protein A (RPA) are the major sensor proteins that detect DSBs and ssDNA, respectively [24–27]. These proteins then recruit ATM (ataxia-telangiectasia mutated) and ATR (ATM- and Rad3-related), both of which are the main kinases of the DDR [25]. ATM is largely engaged in DSB repair, whereas ATR is primarily involved in the recognition of ssDNA wrapped by RPA [26] (Fig. 1). Although ATM and ATR recognize distinct forms of DNA damage, both are needed for proper checkpoint activation when DSBs are encountered [27–30]. The cis-local phosphorylation of histone H2AX (γ-H2AX) by ATM and ATR is a critical step in DDR [31]. MDC1 (mediator of DNA damage checkpoint protein 1) is hyperphosphorylated in an ATM-dependent manner, generating a phospho-specific domain that can detect γH2AX [32–34]. MDC1 recruitment to γH2AX amplified local ATM activity and the spreading of γH2AX along the chromatin from the DSB. This in turn raises the local concentration of many DDR components at the site of DNA damage, resulting in a positive feedback loop that amplifies ATM activity [7, 35–37]. Co-localization of ATRIP (ATR interaction protein) [39] and the 9-1-1 complex (composed of RAD9, RAD1, and HUS1) [40] is also required for ATR activation by RPA-coated ssDNA [38, 39]. Furthermore, topoisomerase II binding protein 1 (TOPBP1) is an ATR signaling pathway amplifier [40, 41] (Fig. 1).

## Regulation of cell cycle progression by DDR

DNA damage signaling activates cell cycle checkpoints, halting cell cycle advancement and allowing time for DNA repair, preventing damaged DNA replication. The cell cycle is divided into four stages: G1, S, G2, and M, each with its own set of regulatory proteins. Cyclin D, CDK4/6, and p16INK4a are examples of G1 phase regulatory proteins, whereas cyclin E, CDK2, and p21 are examples of S phase regulatory proteins. The production of cyclin E and CDK2 complexes is required for cells to enter S phase; G2 phase regulatory proteins primarily involve cyclin B and CDK1, and the formation of a complex between the two causes cells to enter pre-M phase [42–44].

Activated ATM and ATR phosphorylate the activating checkpoint kinases CHK2 and CHK1, with ATR primarily activating CHK1 but also ATM [45, 46]. Activated CHK1 and CHK2 then phosphorylate the cell division cycle 25 (CDC25) phosphatase and the tumor suppressor protein p53, causing their inactivation or degradation and activation or stabilization, respectively [47–50]. Furthermore, active CHK1 in yeast stimulates Wee1 kinase, which inactivates CDK1 and CycB [51, 52]. Both eventually produce cell cycle arrest: the ATM-CHK2-P53 pathway regulates the G1 checkpoint, while the ATR-CHK1 pathway controls the S and G2/M checkpoints [42, 53], and both pathways can cause cell cycle arrest through p53 activation (Fig. 1 upper panel). p53 is a transcription factor that regulates genes involved in DNA repair, cell cycle arrest, apoptosis, and metabolism [54, 55]. Phosphorylated P53 promotes the expression of the cyclin-dependent kinase inhibitor (CDKI) p21. Both p21 and p16 cyclin-dependent kinase inhibitors are components of the tumor suppressor pathway and a major modulator of senescence-associated cell cycle arrest. CycE and CDK2 are inhibited by p21 activation. CycD and cdk4/6 cyclins are inhibited by p16 activation. The mechanism of p16 induction remains unknown [56]. Furthermore, both p21 and p16 can keep the retinoblastoma protein (pRB) hypophosphorylated and active, resulting in cellular senescence [57].

## Repair mechanisms in the DNA damage response

The primary repair pathways for DSBs are non-homologous end-joining (NHEJ) and homologous recombination (HR). Non-homologous end joining (NHEJ) re-ligates a DSB without extensive processing of the DNA around the DSB and is present throughout the cell cycle, making it a relatively easier, faster, and more extensive repair mechanism of the two. HR, on the other hand, necessitates resection of the DNA at the break site to form substantial single-stranded overhangs that can invade the homologous sister strand, which is more difficult and precise and occurs only in the S/G2 phase [6, 58]. On the DSB, γH2AX progressively recruits MDC1, RNF8, and RNF168, triggering a ubiquitination cascade around the DSB [59]. Following this, the DSB repair proteins BRCA1 and 53BP1 are recruited [59, 60]. The 53BP1-RIF1 and the BRCA1-CtIP pathway are in competition with each other and their selection is regulated by the cell cycle and histone modifications [44, 61, 62]. In the G1 phase, the recruitment of 53BP1-RIF1 enhances NHEJ repair by antagonizing the recruitment of the BRCA1-CtIP complex [44]. In the S/G2 phase, CtIP cooperates with nucleases to produce extensive single-stranded overhangs by excising DNA at the break site and invading the sister homologous strand. During this process, exposed ssDNA is first bound by RPA [6], and then the recombinase RDA51 displaces RPA in the involvement of recombinant mediators BRCA1, PALB2, and BRCA2 to form RAD51-single-stranded DNA nucleoprotein filaments. This nucleoprotein filament structure is capable of facilitating multiple processes such as homology search, strand invasion, and DNA polymerization [44, 63, 64] (Fig. 1 lower panel).

## The link between cellular senescence and DNA damage

Cellular senescence is a state of irreversible cell cycle arrest. Cellular senescence can be caused by a variety of factors, including telomere malfunction, DNA damage, oncogene activation, and organelle stress [5, 204]. DNA damage is likely the most powerful cause of cellular senescence, as DNA carries information about all of the proteins and RNAs produced by the cell [65, 66]. If DNA damage cannot be repaired and continues, it can result in prolonged DDR signaling and long-term proliferation arrest in the form of cellular senescence [48]. DDR foci harboring unrepaired DSBs have been reported in cultured senescent cells [49]. Inhibiting DDR signaling kinases (ATM, ATR, CHK1, and CHK2) permits senescent cells to re-enter the cell cycle [67–69]. Furthermore, even in the absence of physical DNA damage, alterations in DDR sensors alone can cause cell cycle arrest [70].

**Fig. 2:**
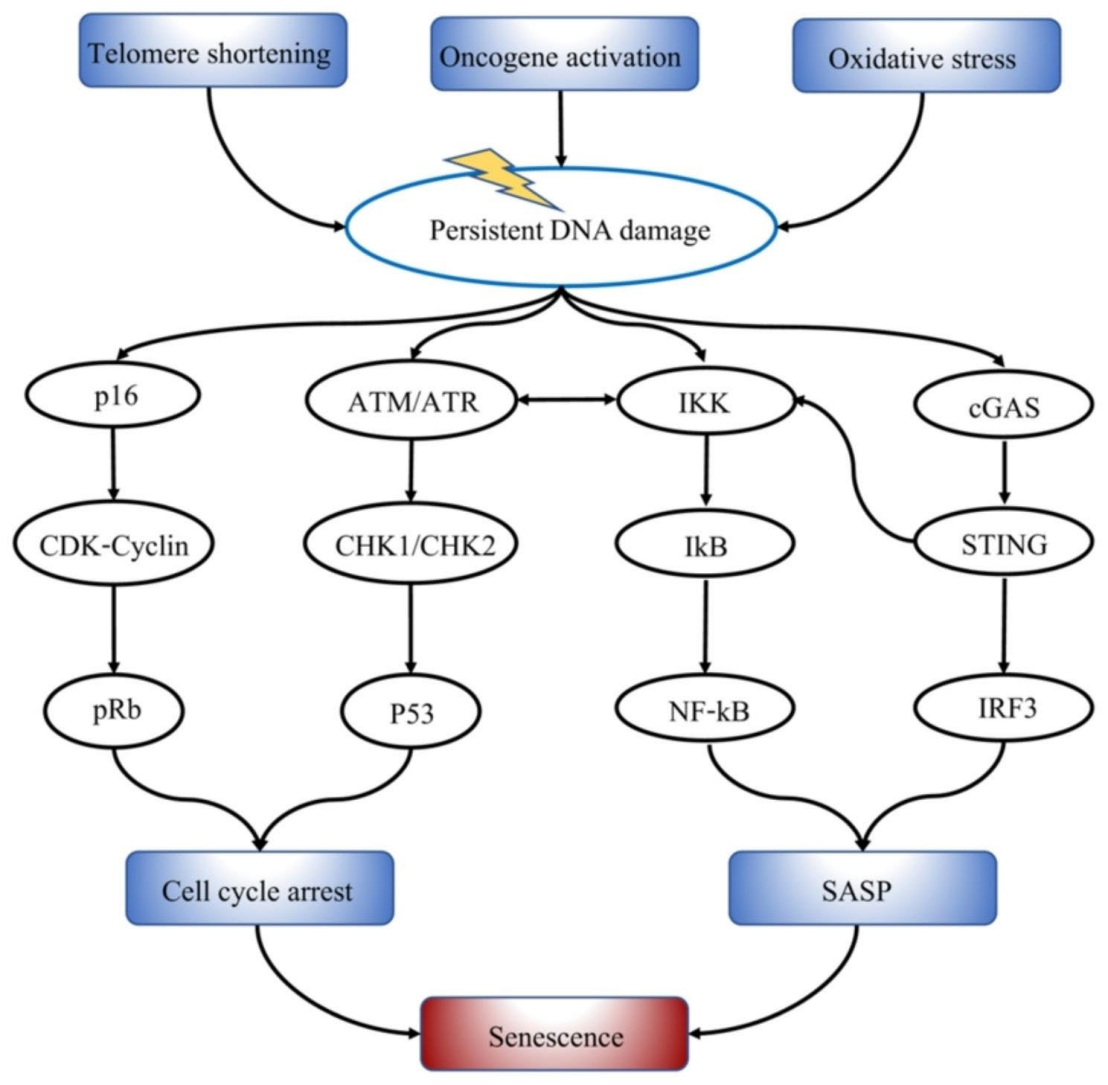
The relationship between cellular senescence and DNA damage. Senescence can be activated by different stimuli, including telomere shortening, DNA damage, oxidative stress, and oncogene activation. A central factor involved in all aspects of senescence is the sustained DNA damage response (DDR), which causes cell cycle arrest via the p53 and RB-dependent pathways and SASP secretion via the NF-kB and cGAS-STING pathways, ultimately inducing cellular senescence. This figure was modified according to the published Fig. 1 in reference [204].

Cellular senescence was initially identified as the mechanism that regulates the limited replicative lifespan of cultivated cells, also known as replicative senescence (RS) [2], a type of telomere-induced cellular senescence (TIS). Telomeres shorten with each round of DNA replication due to a lack of telomere maintenance mechanisms like telomerase expression or telomere recombination. Such ends are regarded as double-strand breaks (DSBs) below a specific length, triggering a DNA damage response (DDR) [68, 71]. However, aberrant activation of the proliferative pathway can also cause cellular senescence. Oncogene-induced senescence (OIS) is characterized by substantial activation of the DDR pathway and the formation of DDR foci in senescent cells (also known as senescence-associated DNA-damage foci; SDFs) [7, 67, 72, 73]. Furthermore, mitochondrial dysfunction induces increased ROS generation in senescent cells, resulting in DNA damage and DDR activation [74, 75], which drives cellular senescence [76, 77]. Clearly, all of these senescence-inducing conditions influence DDR, which plays a critical role in cellular senescence (Fig. 2).

**Fig. 3:**
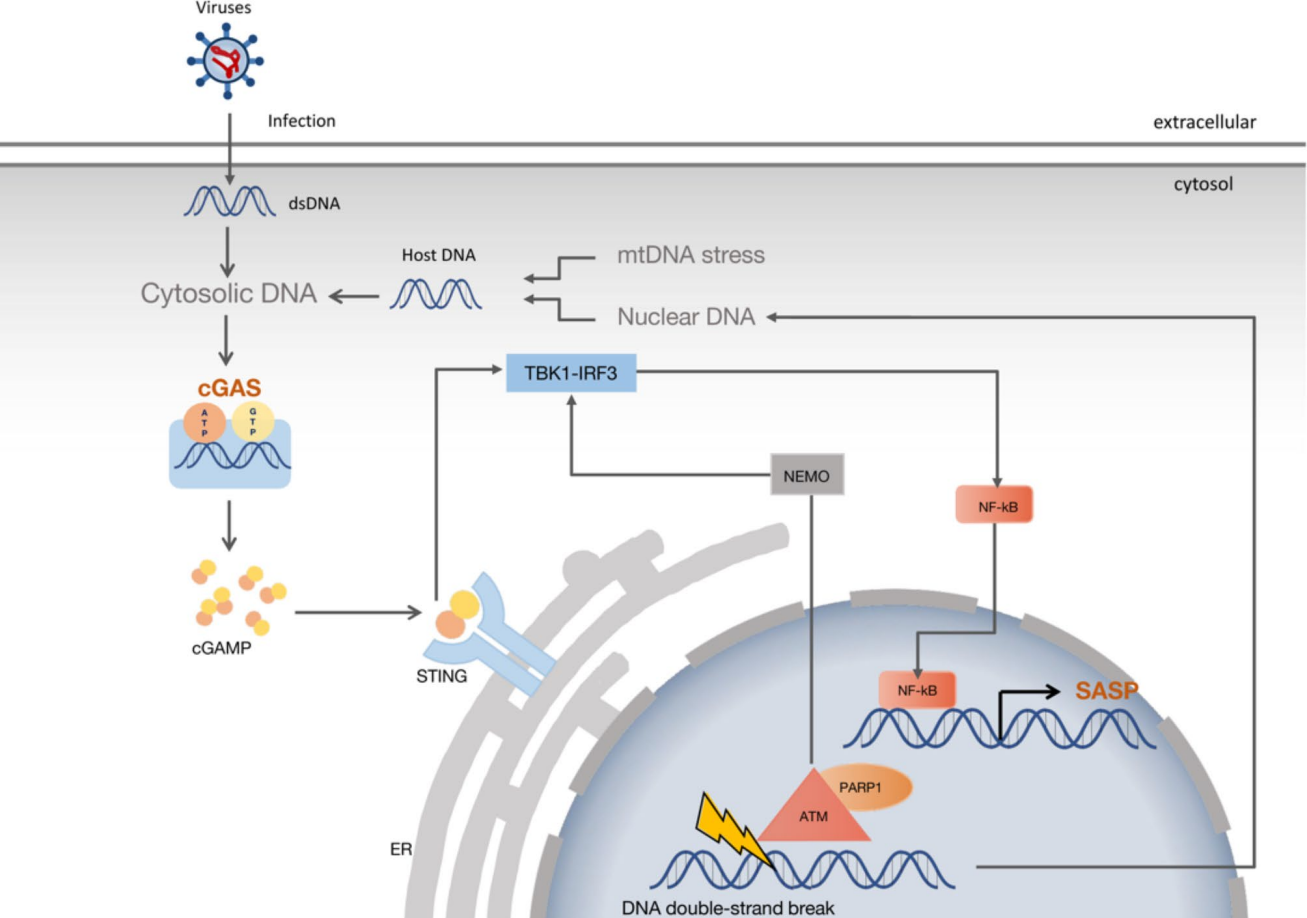
cGAS is a key linkage between DNA damage and SASP. Exogenous and Endogenous aberrant DNA bind cyclic GMP-AMP synthase (cGAS) and activate the synthesis of 2′3′-cyclic GMP-AMP (2′3′-cGAMP), which binds to and induces oligomerization of STING (stimulator of interferon genes) in the endoplasmic reticulum and its incorporation into vesicles. When STING is activated, it attracts and activates TANK-binding kinase 1 (TBK1), which phosphorylates STING and the interferon regulatory factor IRF3, activating the NF-κB signaling cascade. The sensor kinase ataxia telangiectasia mutated (ATM) also activates TBK1, through the phosphorylation of NF-κB essential modulator (NEMO), a member of the IB kinase complex that activates NF-κB. In response to nuclear DNA damage, ATM can potentially activate STING in a non-canonical manner. PARP-1, poly (ADP-ribose) polymerase 1, is an essential DNA damage sensor. This figure was modified according to the published Fig. 3 in reference [202].

Furthermore, cellular senescence is frequently regarded as a stress response that, in addition to the characteristic stable cell cycle arrest, involves a pro-inflammatory phenotype known as the senescence-associated secretory phenotype (SASP), which is primarily mediated by the cGAS-STING, NF-κB, and C/EBPβ signaling pathways [3, 78]. Studies have shown that the gene expression of SASP often requires sustained DDR signaling and that key DDR proteins such as ATM, NBS1, and CHK2 are involved in the activation of SASP genes [78, 79] (Fig. 3). Defective DDR signaling is a fundamental mechanism of DNA damage, cellular senescence, and aging [80].

Cyclic GMP-AMP synthase (cGAS) has been found to be a key linkage between DNA damage, SASP gene expression, and cellular senescence [81]. SASP gene expression is reduced when cGAS is deleted [81]. cGAS or stimulator of interferon genes (STING) deprived cells are unable to induce senescence by DNA damage stimulation, and p16, p21, and SASP are also not increased [81–83]. The binding of cGAS to cytoplasmic dsDNA fragments, including double-stranded DNA from the leaky senescent nuclei and exogenous nucleic acids (viruses), initiates the cGAS-STING pathway [202, 84, 85] (Fig. 3). Activated cGAS catalyzes the formation of cyclized dinucleotides (cGAMP) from ATP and GTP. cGAMP translocates to the endoplasmic reticulum, where it binds to and activates STING [84, 86]. Activated STING translocates to the Golgi apparatus and recruits TANK-binding kinase 1 (TBK1) and IκB kinase (IKK), which activate Interferon regulatory factor 3 (IRF3) and NF-kB [86–88].In most unstimulated cells, NF-kB dimers are found in the cytoplasm as complexes with IkB proteins. Upon stimulation, IkB is phosphorylated by the IKK complex, ubiquitinated, and targeted for degradation, thus releasing the NF-kB subunits that translocate to the nucleus and induce transcription of inflammatory proteins like type I interferon [86–89]. Direct activation of the NF-kB signaling pathway by nuclear DNA damage necessitates the activation of ATM and PARP1 [43, 90], resulting in the phosphorylation and ubiquitination of sumoylated NEMO. PARP-1 is an essential DNA damage sensor [91]. NEMO is a regulatory subunit of the IκB kinase complex. Ubiquitinated NEMO coupled with ATM is exported into the cytoplasm, where it activates the IKK complex and then the NF-kB signaling cascade like the traditional pathway [90, 92].

## Cellular senescence Induced by infection with HCMV and other viruses

**Fig. 4:**
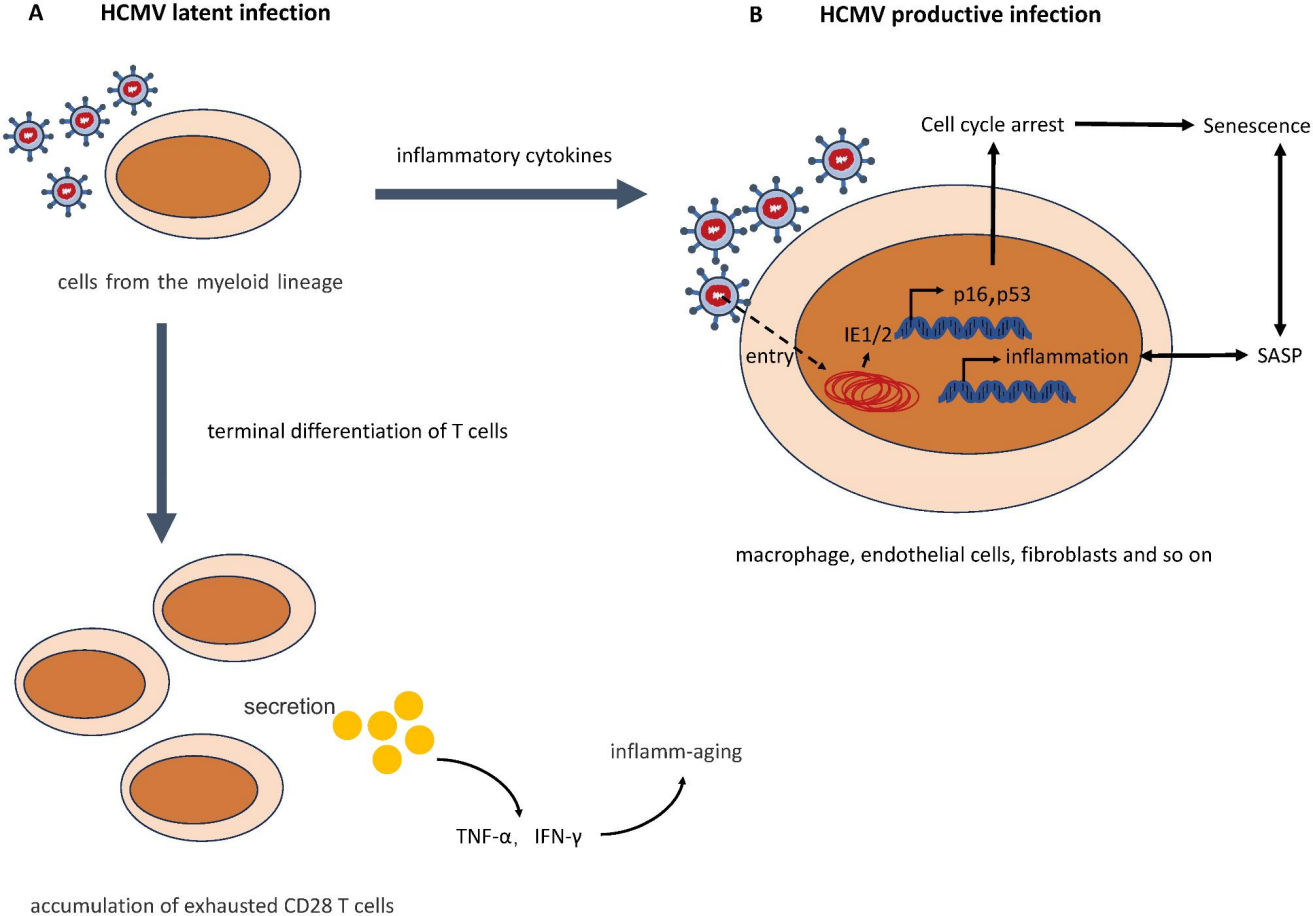
The model of HCMV-induced senescence. Human cytomegalovirus (HCMV) has two modes of infection: latent and productive. (**A**) In the latent infection, cells from the myeloid lineage are considered as primary sites. Long exposure to HCMV enables terminal differentiation of T cells leading to accumulation of exhausted CD28 T cells, which secrete TNF-α and IFN-γ to promote inflamm-aging. (**B**) In the productive infection, HCMV-infected cells show senescence phenotype, including cell cycle arrest and SASP.

Immature myeloid lineage cells present in the bone marrow and circulating in the blood are considered as primary sites for viral latency [93–96]. Although persistent CMV infection is systemically controlled by the immune system and viral particles are detectable only in times of reactivation, life-long exposure to HCMV has been demonstrated to severely impair the T cell system. It increases the number of highly differentiated, exhausted CD4 and CD8 T cells, named terminally differentiated T Cells [97, 98]. One of the most robust markers in describing these exhausted T cells is the lack of the costimulatory molecule CD28, a member of the tumor necrosis factor receptor family that interacts with CD80 and/or CD86 expressed on activated antigen-presenting cells [99]. The age-dependent accumulation of exhausted CD28^+^ T cells, which preferentially produce the pro-inflammatory cytokines IFN-γ and TNF-α, is thought to contribute—together with components of the innate immune system—to the low-grade pro-inflammatory background observed in elderly persons (inflamm-aging) [100] (Fig. 4A).

Cells from the myeloid lineage are thought to play a critical role in HCMV latency and reactivation but do not support productive infection [93–95]. Instead, this virus can infect many other cell types, in most of which virus causes a productive infection, such as macrophage, endothelial cells, fibroblasts [101, 102]. Complex mechanisms control the reactivation of the HCMV from latency. Inflammation has been shown to have the potential to cause latent HCMV to reactivate [103–105] (Fig. 4).

Tracking HCMV infection with single-cell transcriptomics revealed that infection outcome (productive or latent) is also based on viral gene expression levels at early stages of infection [106]. High early viral gene expression levels, particularly of immediate early (IE) genes, facilitate productive infection [106, 107]. In the productive infection, previous reports demonstrate that HCMV induces premature senescence in early passage human fibroblasts, similar to senescent cells which have reached the limit of their replicative capacity [108]. Specifically, the IE1 protein activates and interacts with p53, causing p53 accumulation [109, 110]. The IE2 protein inhibits cellular DNA synthesis, resulting in cell cycle arrest through a functional p53 pathway [111]. The interaction of IE1, IE2, and p53 above ultimately evokes the senescence phenotype in HCMV-infected cells [16, 110, 111]. Additionally, HCMV infection upregulates the expression of p16, which is necessary for ideal viral replication [112]. Furthermore, HCMV infection affects the inflammatory phenotype in addition to causing cell cycle arrest [17] (Fig. 4B).

According to recent researches, virus infections, such as measles virus, human respiratory syncytial virus and COVID-19, can prematurely stimulate cellular senescence, known as virus-induced senescence (VIS) [23, 113, 114]. Measles virus (MV) infection has been proven to induce p53 and p16-pRb pathway-dependent cellular senescence *via* cell [115]. Epstein–Barr virus (EBV), Kaposi sarcoma herpesvirus (KSHV) and human respiratory syncytial virus (RSV) infections can trigger DNA damage-mediated cellular senescence through replicative stress or induction of mitochondrial ROS [23, 116, 117]. Senescence markers and SASP factors have been found in tissue samples of the nasopharyngeal cavity and lungs of patients suffering from coronavirus disease 2019 (COVID-19) with severe disease progression [20]. A basic research study, assessing the occurrence of VIS, found that human diploid fibroblast models exposed to high-titer retrovirus exhibited typical senescence and the activated cyclic GMP-AMP synthase-stimulator of interferon genes (cGAS-STING) pathway after the fifth day of infection [118].

## HCMV infection can promote cellular senescence by modulating the DDR

As mentioned above, Virus-induced senescence (VIS) has been a widespread event [20]. Viral infections generate a variety of cellular impairments, including DNA damage [23], as well as significant biological changes in host cells, such as cellular senescence [23, 115, 119]. Similarly, like activators of DNA damage, oncogenes [120–122] and oxidative stress [123–125], we speculate that DDR plays a key role in cellular senescence induced by infection with HCMV.

After penetration of the plasma membrane, components of the virion, including its 240-kb linear double-stranded DNA (dsDNA) genome (which consists of two unique coding sequences [U_L_ and U_S_] flanked by a series of inverted repeat, are rapidly transported to the nucleus, where viral transcription and replication take place [21, 126]. It has been proved that the entrance of the HCMV genome into the infected cell nucleus can initiate DDR during productive infection [21–23].

## HCMV is a DNA damage-inducing factor

HCMV infection is genotoxic to host cells, and the type and quantity of damage rely on viral genome expression and the cell cycle phase at the time of viral infection [127]. Infected host cells cause particular breaks on chromosome 1, 1q42 and 1q21, during the S phase [128]. Stably transfected cells expressing HCMV UL76 develop chromosome aberrations including micronuclei and misaligned chromosomes, lagging and bridging, and activate the DNA damage signal γH2AX, causing foci formation in nuclei [129]. HCMV infection interfering with cellular replication can induce replication stress (RS) with ensuing implications for genomic integrity. In addition, expression of IE1 and IE2, driven by the viral major immediate early enhancer and promoter (MIEP), has been determined to induce RS alone [130].

Furthermore, there is accumulating evidence that viral infection can generate oxidative stress [131–133], which can lead to DNA damage [74, 75]. HCMV infection has been found to increase ROS generation [18] and mitochondrial biogenesis [134]. ROS promotes HCMV replication via paracrine and autocrine pathways, and N-acetylcysteine, a ubiquitous H_2_O_2_ scavenger, decreases HCMV replication activation [135]. Interestingly, HCMV appears to utilize virus-specific mechanisms to protect the cells from the harmful effects of ROS and maintain redox homeostasis [125]. There is no doubt that HCMV and ROS have a complementary relationship, and there is evidence that both HCMV and ROS can cause DNA damage, but there is still no direct evidence that HCMV-mediated increase in ROS leads to DNA damage, which would be interesting to investigate.

## HCMV influences cell cycle checkpoint activation during DDR

Human cytomegalovirus (HCMV) infection activates multiple DDR proteins, including ATM and downstream effector proteins p53 and H2AX [21, 110, 136]. These proteins are also necessary for efficient HCMV replication [137, 138]. Activated p53 directly induces p21 [139], ultimately leading to cell cycle arrest in HCMV-infected fibroblasts [138, 140]. Immediate early 1 (IE1) of HCMV is an important viral protein for the induction of DDR. Its stimulation of cellular DDR was first described by Castillo et al., who showed that IE1 was sufficient to activate ATM. ATM subsequently activates the p53 pathway by phosphorylation [110]. This conclusion was later supported by additional research, which also showed that the DSB marker γH2AX is similarly activated in an IE1-dependent way [137]. In addition to the activation of ATM by IE1, HCMV infection also leads to ATM autophosphorylation [136]. Interestingly, p53 is bound by IE2 but its transactivation activity is inhibited [141, 142]。.

**Fig. 5:**
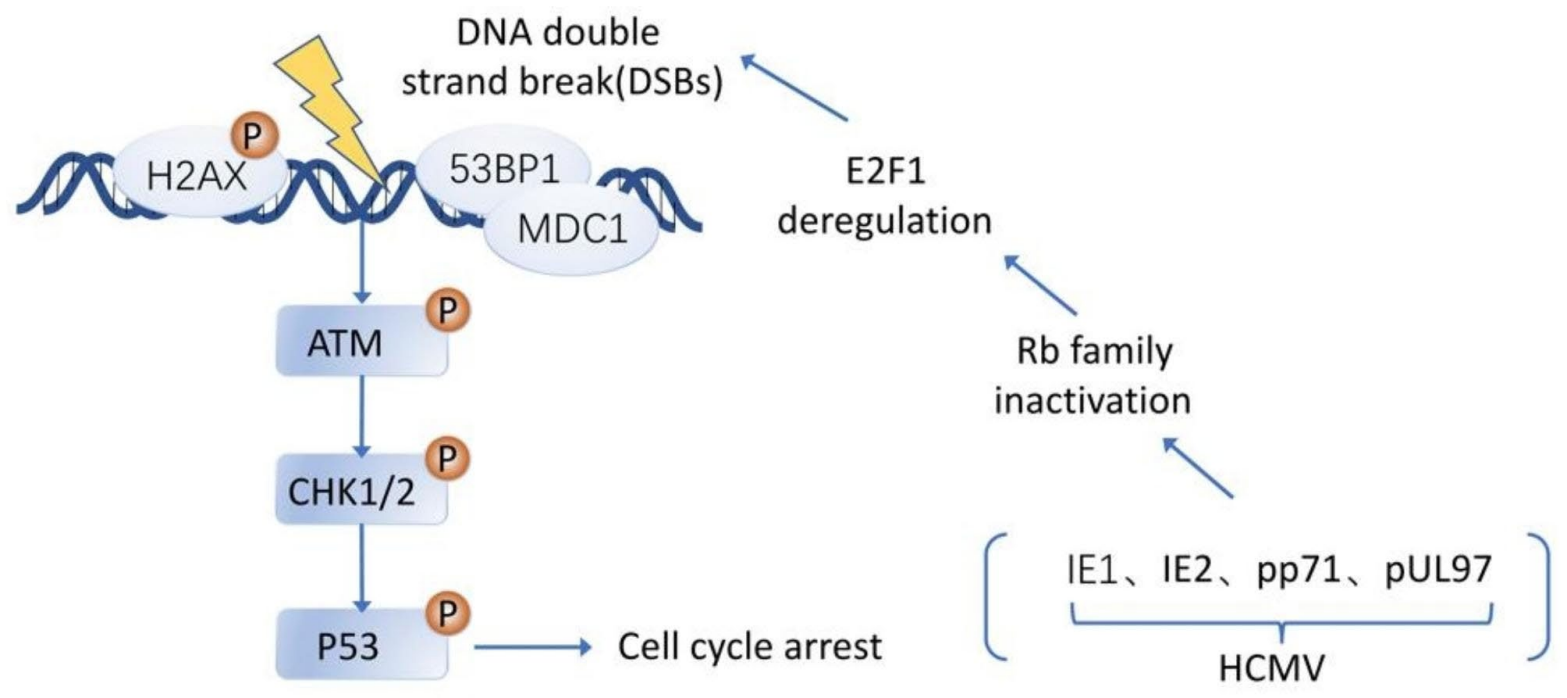
Model of the host DNA damage response induced by HCMV infection. Efficient replication of HCMV requires DDR centered on ATM and E2F1. HCMV infection can activate multiple DDR proteins, including ATM and the downstream effector proteins p53 and H2AX. IE1, IE2, pp71, and pUL97 of HCMV can inactivate RB family members, leading to dysregulation of E2F1 proteins and subsequent production of DSBs.

Efficient HCMV replication requires a host DDR that centers on the presence of ATM and E2F1 protein [137]. E2F1 is a protein in the E2F family that belongs to the RB-regulated activator class [143, 144]. It has been shown that RB inactivation and deregulation of E2F1 leads to DNA double-strand break (DSB) accumulation and cell cycle checkpoint signaling [145–148] (Fig. 5). One of the earliest impacts of HCMV infection has been identified as RB family protein inactivation [137]. IE1, IE2, pp71, and pUL97 of HCMV, all of which can inactivate RB family members [15, 149–156], lead to dysregulation of E2F1 proteins and induction of DSBs [146]. And the resulting activation of ATM and its downstream target phosphorylation, including H2AX and p53, contribute to the replication of HCMV and cell cycle arrest in the host cell [137, 157].

However, the role of DDR in HCMV replication has long been controversial. Although ATM is important for virus replication in cells [137], HCMV replication in cells lacking ATM has also been reported [21], Some DDR proteins have been shown to mislocate from the nucleus to the cytoplasm after infection, blocking checkpoint signaling and inhibiting host DDR. Therefore, HCMV is also able to escape some of the consequences produced by DDR [158, 159]. In conclusion, ATM and ATR control multiple pathways, and more research are needed to elucidate how HCMV targets DDR and which specific components are regulated by HCMV.

## HCMV infection affects damage repair mechanisms during DDR

The replication of the human cytomegalovirus (HCMV) genome is assumed to be biphasic [160]. The initial phase of infection is characterized by origin-specific replication from the input circularized genome, which leads to single copies of the virus. Later, replication switches to a rolling loop process, resulting in the formation of huge concentric circles [157, 161]. HR happens along conjugated DNA, as indicated by the inversion of genomic sequences in contiguous monomeric units. HR occurs between freely cleaved monomeric and conjugated structures as well, with intermediate structures forming branches late in the infection period [162–165]. It has been suggested that these recombinant structures trigger a DNA damage response (DDR) in host cells during herpesvirus replication [21, 158, 166, 167]. Previous research has also revealed that viral proteins can govern HR [168–170].

The IE1 protein of HCMV is not only a strong activator of DDR, but it can also accelerate HR [110, 137, 168]. Further research found that the IE1 protein, in a novel way, can activate flap endonuclease 1 (FEN1), a cellular factor recently identified to be involved in HR-mediated repair of stalled replication forks by actively inducing DSBs [171], hence restarting stalled replication forks in viral replication [172]. Furthermore, IE1 binds to p53 and inhibits p53’s inhibitory impact on Rad51, enabling HR [173]. Rad51 is a key regulator of HR, and its levels are much higher in HCMV-infected human foreskin fibroblasts (HFFs) [21], but not in normal cells [174]. We hypothesize that viruses may employ the cellular HDR process to boost the efficiency and fidelity of viral genome replication [160, 175, 176].

## HCMV infection regulates SASP secretion

IL-8 and IL-6 are important SASP factors that participate in HCMV infection. HCMV UL76 protein can activate the NF-kB system via the DDR, thereby inducing IL-8 expression [92] and enhancing HCMV replication [177]. As a crucial part of SASP, IL8 activates the chemokine receptor CXCR2 (IL8RB), enhancing DDR and promoting replicative senescence(RS) and oncogene-induced senescence (OIS) [178, 179]. US28, a G protein–coupled receptor encoded by HCMV promotes the production of interleukin-6 (IL-6) [180, 181], whose depletion would cause the inflammatory network to collapse and abolished senescence entry and maintenance [182].

In addition, it has been proved that cellular senescence was induced in host cells upon HCMV infection [81], which was recognized as an antiviral immune response [113, 119, 183]. Mechanistically, this induction of cellular senescence was mainly due to activation of the cGAS-STING pathway triggered by HCMV dsDNA as well as the subsequent SASP secretion [85, 184]. Interestingly, it has been demonstrated that HCMV has evolved multiple strategies to antagonize the activation of GAS-STING signaling in host cells. UL31 and UL42 interacted with cGAS respectively, inhibiting DNA binding and enzymatic activity of cGAS [185, 186]; pp65 selectively bound to cGAS and prevented its interaction with STING, thus inactivating the signaling pathway through the cGAS/STING/IRF3 axis [187]. UL82, UL94 and US9 interacted with STING respectively, disrupting the translocation of STING and impairing the TBK1 recruitment to the STING signalsome [188–191]; pUL48 had a ubiquitinating effect on STING and IE2 protein facilitated the proteasome-dependent degradation of STING, both of them inhibiting STING-induced IFN-β promoter activation [192, 193]; UL35 and UL37 × 1 downmodulated this signaling pathway at the level of the key signaling factor TBK1 [194, 195]; UL138 inhibited the pathway downstream of STING but upstream of IRF3 phosphorylation and NF-κB function [196]. Although the cGAS-STING signaling induced by HCMV dsDNA was challenged by the HCMV encoded inhibitors described above serving for the viral immune escape, this pathway remained activated [184] and subsequently induced cellular senescence [81].

Previous research has demonstrated that HCMV-infected fibroblasts can mimic senescence-associated inflammation and elicit a significant inflammatory response, potentially leading to the development of age-related inflammatory disorders [17]. As a result, we hypothesize that DDR is intrinsically connected to HCMV-induced SASP production and cellular senescence.

## Conclusion

Previous studies have shown that HCMV infection triggers molecular mechanisms associated with host cell senescence [16, 109–112] as well as inflammatory responses [17, 184, 197]. However, there is little evidence to explain why HCMV can cause senescence-associated phenotypes in host cells. A growing number of studies demonstrate that HCMV might alter the DNA damage response (DDR), for example, by acting directly as a DNA damage agent, interacting with essential DDR proteins, and activating the cGAS itself as aberrant DNA [21, 110, 136]. As a result, we argue that DDR may be one of the reasons why HCMV can generate the senescence phenotype.

Interestingly, cellular senescence has been proposed as a key mechanism of viral invasion resistance [183]. Viral entrance generates major biological changes in infected host cells as a viral-triggered state shift that may lead to cellular senescence [23, 115], with varied degrees of impact on virus proliferation [119, 198]. Stable cell cycle stoppage and the release of pro-inflammatory cytokines and chemokines associated with cellular senescence may give rise to antiviral response features [119]. Leading to speculation that cellular senescence may have evolved as an antiviral defense mechanism [183, 199]. This notion is strengthened by the function of endogenous IFN-b, which is generated by DNA damage, in the induction of senescence [200]. Surprisingly, recent researches highlight a commensal-like function for HCMV in the immunosurveillance of aging cells in immunocompetent hosts: on the one hand, HCMV can be reactivated in senescent fibroblasts, but with low IE1/2 expression and the absence of productive infection, and on the other hand, CD4 CTLs are able to target HCMV-gB antigens to recognize and clear senescent cells [119, 183, 201].

In conclusion, the significance of HCMV in the aging process is receiving increased attention and is intricately related to all aspects of aging. Here we focus on the effects of HCMV on cellular senescence. As to how HCMV causes cellular senescence, there are necessarily many other mechanisms involved besides DDR, and more research is needed to demonstrate this.

## Data Availability

Not applicable.

## Abbreviations

ATM: Ataxia-telangiectasia mutated
ATR: ATM- and Rad3-related
CDC25: Cell division cycle 25
cGAS: Cyclic GMP-AMP synthase
DDR: The DNA damage response
DSBs: DNA double-strand breaks
HCMV: Human cytomegalovirus
HR: Homologous recombination
IKK: IκB kinase
MDC1: Mediator of DNA damage checkpoint protein 1
MRN: MRE11-RAD50-NBS1
NHEJ: Non-homologous end-joining
RPA: Replication protein A
SASP: Senescence-associated secretory phenotype
ssDNA: Single-stranded DNA
STING: Stimulator of interferon genes
TBK1: TANK-binding kinase 1
TOPBP1: Topoisomerase II binding protein 1
VIS: virus-induced senescence
